# Interactions of boron released from surface pre-reacted glass ionomer with enamel/dentin and its effect on pH

**DOI:** 10.1038/s41598-021-95279-x

**Published:** 2021-08-03

**Authors:** Noriko Hiraishi, Mahmoud Sayed, Robert Hill, Junji Tagami, Fumiaki Hayashi

**Affiliations:** 1 Cariology and Operative Dentistry, Graduate School of Medical and Dental Sciences, Tokyo Medical and Dental University, 1-5-45, Yushima, Bunkyo-ku, Tokyo, 113-8549 Japan; 2 Dental Physical Sciences, Barts and the London School of Medicine and Dentistry, Institute of Dentistry, Queen Mary University of London, London, UK; 3 Advanced NMR Application and Platform Team, NMR Research and Collaboration Group, NMR Science and Development Division, RIKEN SPring-8 Center, Hyogo, Japan

**Keywords:** Preventive dentistry, Restorative dentistry

## Abstract

This study investigated the interaction of borate ions released from surface pre-reacted glass ionomer (S-PRG) fillers with the biological apatites of enamel and dentin using solid-state (SS) magic-angle spinning nuclear magnetic resonance (MAS-NMR) spectroscopy analysis. We further evaluated the effect of borate ions on the pH change. Bovine enamel and dentin powder were submerged in S-PRG eluate (with borate ion concentration of 100 mM) for 3 h, then washed with deionized water (DW). The dried enamel and dentin specimens were used for ^11^B SS-NMR and Fourier transform infrared spectroscopy (FT-IR) analysis. Enamel and dentin blocks were treated with three solutions: DW (control), S-PRG eluent and borate solution (100 mM). The treated samples were submerged in the demineralization solution and the pH was measured using a pH meter daily for 6 days. The surfaces of the enamel and dentin blocks were then observed using field emission scanning electron microscopy (FE-SEM). SS-NMR analysis revealed the presence of adsorbed borate ions in the enamel and dentin samples in a tetra-coordinated form. The pH results demonstrated an increase in pH values in the S-PRG and borate groups. SEM images showed that the surfaces of the control group were demineralized, whereas the surfaces of the S-PRG and borate groups were intact. These results concluded that borate ions could be adsorbed to enamel and dentin in the tetra-coordinated form. Borate ions possess a buffer capacity which may help to protect the tooth structure against acid attacks and promote remineralization.

## Introduction

Despite the availability of numerous fluoride interventions, dental caries remains a common chronic disease with a global prevalence of 35%^1^. Therefore, researchers have been investigating other strategies which work synergistically with or better than fluoride to reduce the risk of dental caries^2^.

Borate-based bioactive glasses are highly biocompatible materials with a wide variety of uses in medical and dental fields. Borate bioactive glasses are widely known to have various abilities, such as supporting the growth of bone cells^3^ and promoting the formation of hydroxyapatite (HAp)^4^, as well as strongly bonding to hard and soft tissues^5^. Bioactive glasses have numerous biomedical applications, including the repair of bone defects, scaffolds for bone and tooth tissue engineering, and teeth remineralization^6^.

Among the innovations which promote the concept of preventive and minimal invasive management of dental caries is the three-layer bioactive, Surface Pre-Reacted Glass ionomer filler (S-PRG)^7^. S-PRG filler is a unique particle which has been developed with the pre-reacted glass-ionomer technology to impart bioactive functions to restorative materials^8^. The pre-reacted glass-ionomer phase on the surface of the glass core allows S-PRG filler to release and recharge fluoride ions^9^. Moreover, the S-PRG filler has a unique characteristic of releasing other ions, such as aluminum (Al^3+^), borate (BO_3_^3−^), sodium (Na^+^), silicate (SiO_4_^4−^), and strontium (Sr^2+^) ions, since a fluoro-boro-alumino-silicate glass is used as the glass core^10, 11^.

Studies have reported that various bioactive effects are associated with the ions which are released from S-PRG, including antibacterial effects^12^, prevention of demineralization^13^, and enhancement of remineralization^14^. BO_3_^3−^ ion is well known for its antibacterial effect^15^. In addition, borate ions have biological effects, such as promoting bone formation and increasing HAp formation^4, 16^.

Previous studies investigated the incorporation of ions quantitatively, which were released from S-PRG fillers into the tooth structure, using electron probe micro analysis (EPMA)^17^, particle-induced X-ray (γ-ray) emission (PIXE/PIGE)^18^ and inductively coupled plasma atomic emission spectroscopy (ICP-AES)^19^. However, the chemical state and reactions of borate ion with the biological HAp of tooth structure or its effects on pH were not discussed earlier.

NMR can provide information at the atomic level about the structure and crystallinity for any material^20^. ^11^B solid-state NMR is helpful to characterize of boron-containing compounds and can detect all structural forms of boron which exist with any compound^21^.

This study aimed to investigate the chemical interaction outcome of borate ions released from S-PRG fillers with the biological apatites of enamel and dentin using solid-state MAS-NMR spectroscopy analysis. Furthermore, we evaluated the effect of borate ions on the change of pH and observed the changes in the surface topography using FE-SEM.

## Materials and methods

This study protocol was approved by the ethics committee of Tokyo Medical and Dental University under identification code “D2016-090”. All methods in this study were performed following the relevant guidelines and regulations.

Pulverized dentin (0.84 g) and enamel (0.6 g) powder with particle size 50–75 μm were obtained from the crowns of bovine incisor teeth. The crowns of the teeth were sliced into 1 mm thick sections, after which the enamel and dentin portion were carefully separated. The separated enamel and dentin sections were immersed in liquid nitrogen and finely ground to a homogenous powder in a mortar and sieved to 50 ~ 75 μm. Moreover, enamel and dentin blocks (5 × 5 × 1 mm) obtained from bovine incisor teeth were used in this study.

### S-PRG eluate preparation

S-PRG saturated eluate was provided by Shofu Inc, Kyoto, Japan. To simulate the ion release from S-PRG fillers into the water medium, S-PRG saturated eluate was prepared by adding 1 kg of S-PRG fillers to 1 L of deionized water in a tumbler mixer at 23 °C for 24 h to become supernatant. Henceforth, the mixture was filtered through 0.45 μm membrane pore filter. This eluate was measured by Inductively Coupled Plasma to report the ion concentration, as shown in Table 1.

**Table 1:** Different ions concentration in the S-PRG eluate.

pH 7	Al^3+^	BO_3_^3−^	Na^+^	SiO_4_^4−^	Sr^2+^	F^−^
Ion concentration in ppm	18.4	1368.4	477.7	8.4	167.1	127.5
Ion concentration in mM	0.68	126.59	20.78	0.30	1.91	6.71

### NMR specimen preparation and characterization of NMR spectra

The S-PRG saturated eluate was diluted by DW to be 100 mM BO_3_^3−^. This solution was used hereafter as the S-PRG eluate. Each of the dentin (0.28 g), enamel (0.2 g) powder specimens (n = 2) were submerged in the S-PRG eluate (50 mL) (pH 7) for 3 h at 37 °C. In each 50 mL eluate, the mass of dentin specimen was greater than that of enamel specimen to consider the lower mineral percentage of dentin, which was 70% by weight compared to the enamel which has a mineral percentage of 95%^22^. Thereafter, the specimens were washed three times using 50 mL DW. After washing, the treated powder was dried up at room temperature in an ambient atmosphere. Synthetic nanocrystalline hydroxyapatite (nano-HAp) was obtained by the wet chemical technique using Habraken et al. methodology^23^ as follows: the reaction was done in a Tris-buffered saline (TBS) solution containing a 50 mM Trizma-base and 150 mM sodium chloride (NaCl) in DW and the solution was set at pH 7.40. The apatite precipitation was accomplished by incubating 5.88 mM CaCl_2_ with 4.12 mM K_2_HPO_4_ in TBS at 37 °C for 24 h. The specimens were then treated with S-PRG eluate in the same manner for NMR sample. This synthetic apatite served as a comparison with natural biological apatites such as dentin and enamel.

The dehydrated specimens were packed in a 3.2 mm zirconia dioxide rotor for the solid-state NMR measurements. The magic-angle spinning (MAS) frequency was 15 kHz. The ^11^B MAS NMR spectra were acquired in a single-pulse experiment of a 30 s recycle duration using JEOL (ECAII spectrometer 700 MHz). The spectra were acquired for enamel, dentin, nano-HAp and S-PRG fillers with scan accumulation of 256, 160, 868 and 32 scans, respectively. The NMR scans were different between the used substrates as the spectra were acquired with an accumulation between 32 and 868 scans depending on the boron level. The ^11^B NMR spectrum of S-PRG filler was taken to identify the boron coordination as the composition. The S-PRG eluate was also examined to confirm the complexation behavior of borate in aqueous solution. All the data processing was performed using Delta 5.1.3 NMR Software (JEOL, Tokyo, Japan).

### Fourier transform infrared spectroscopy (FTIR)

The dentin (0.28 g) and enamel (0.2 g) powder specimens were prepared in the same method as NMR test. The infrared spectra of the dehydrated specimens in addition to S-PRG fillers were measured by FTIR spectroscopy. Approximately 5 mg of the reacted materials were pressed in a KBr plastic pellet for 1 min at 700 MPa to form a translucent disc for FTIR measurements. The adsorption spectra were measured from 500 to 4000 cm^–1^ with a resolution 4 cm^–1^ using IRT-5200 FTIR spectrometer (ATR pro one, Diamond prisma, JASCO, Tokyo, Japan). The spectra recorded were the average of 32 scans.

### pH measurement

Forty-eight (24 enamel and 24 dentin) specimens (5 × 5 × 1 mm) were used in this test. Enamel samples were obtained from the crown portion of bovine teeth, while dentin specimens were obtained from the cervical portion of bovine root. All specimens were polished till #2000 using silicon carbide (SiC) paper under running water, then sonicated for 5 min in deionized water. A layer of clear acid-resistant nail varnish was applied on the specimen surfaces to expose a window of 5 × 5 mm^2^. Specimens were divided into three groups (n = 8) according to the tested solution applied: deionized water (control), S-PRG eluate and Borate solution (Wako pure, Osaka, Japan) (LOT# PTF6991) with pH 7.4 (adjusted by NaOH) and borate ion concentration of 100 mM. The specimens were submerged in the testing solution (30 mL) for 3 h. Each specimen was then immersed in 5 mL demineralizing solution (50 mM acetic acid, 2.2 mM CaCl_2_ and 2.2 mM KH_2_PO_4_, pH 4.5 adjusted by NaOH)^24^ in a separate plastic chamber for 7 days at 37 °C. The pH was measured daily for 6 days using pH meter (F-52, Horiba ltd., Tokyo, Japan)^25^.

### SEM observation

After finishing pH measurement test, specimens were taken out of the demineralizing solution and washed with DW. After this, specimens were fixed using 2.5% glutaraldehyde for 2 h at 4 °C for primary fixation, followed with 0.1% osmium tetroxide solution for 2 h at 4 °C for secondary fixation then dehydrated in ascending concentrations of ethanol (50%, 70%, 80%, 90% and 95% for 25 min each; then, twice in 100% for 25 min each)^26^. Specimens were then dried in a desiccator and finally sputter-coated with platinum/gold coating. The surfaces were observed using a FE-SEM (FE-SEM, S-4500, Hitachi Ltd., Tokyo, Japan) with operating conditions of 5 kV.

### Statistics

The number of specimens per group (n) was calculated using the sample size determination method for the two tailed t test as follows: n = 2(Z_α/2_ + Z_β_)^2^ × SD^2^/Δ^2^. The significance level was set to 1% (Z_α/2_ = 2.576) and the statistical power level to 90% (Z_β_ = 1.282). The difference between mean pH values obtained after 1 and 2 days (Δ ≈ 0.1) and the standard deviation (SD ≈ 0.03) were obtained from a pilot study with five specimens per group. The sample size calculated using the aforementioned equation was 2.68, however, the number of specimens per group was increased to eight to check the reliability of data. Data were analyzed for normality using the Kolmogorov–Smirnov, and Shapiro–Wilk tests. The pH data were analyzed using two-way repeated measures ANOVA followed by the Turkey-HSD post-hoc test. Significance was set at *p* < 0.05 (n = 8 for each group). Statistical analysis was performed using IBM SPSS (SPSS Inc., IBM Corporation, NY, USA) Statistics Version 23 for Windows.

## Results

### ^11^B MAS-NMR spectra

**Figure 1:**
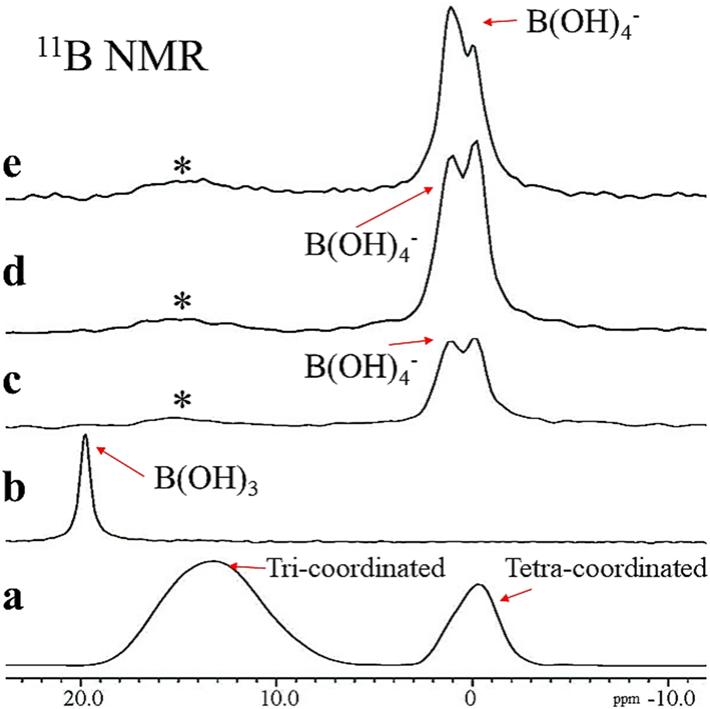
^11^B MAS NMR spectra, (**a**) S-PRG fillers, (**b**) S-PRG eluate, (**c**) S-PRG treated dentin, (**d**) S-PRG treated enamel, (**e**) S-PRG treated nano-HAp.

In the S-PRG fillers specimen (Fig. 1a), two broad spectral peaks were identified at 13.2 ppm and − 0.3 ppm corresponding to tri-coordinated and tetra-coordinated borate ions, respectively^27^. A sharp spectral peak was identified at 20.0 ppm in the S-PRG eluate (Fig. 1b), which is related to aqueous B(OH)_3_. For the enamel, dentin, and nano-HAp powder treated with the S-PRG eluate for 3 h, two dominant peaks, that is, the chemical shifts at 0.0 ppm and 1.0 ppm are attributed to tetra-coordinated borate ion in the form of B(OH)_4_^−^ (Fig. 1c–e). The spectral peaks in dentin and nano-HAp specimens had a higher intensity than enamel specimens. The trace of tri-coordinated borate ion B(OH)_3_ was identified, ranging from 16 to 14 ppm, shown by asterisks shown in Fig. 1c–e.

### FTIR spectra

**Figure 2:**
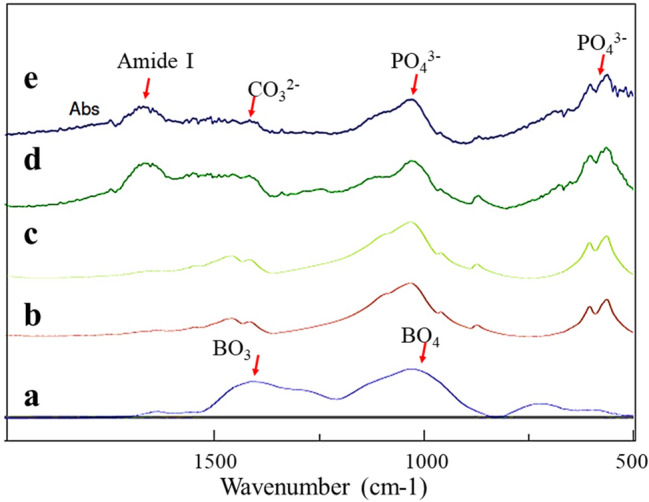
Representative FTIR spectra for the tested specimens, (**a**) S-PRG fillers, (**b**) untreated enamel (control), (**c**) S-PRG treated enamel, (**d**) untreated dentin (control), (**e**) S-PRG treated dentin. The peaks centered at 1067 and 1375 are corresponding to BO_4_ and BO_3,_ respectively.

FTIR spectra were collected for the tested specimens, which is shown in Fig. 2. Both the enamel powder treated with the S-PRG eluate for 3 h and untreated enamel (control), revealed three vibrational band peaks centered at 1032, 603, and 564 cm^−1^ corresponding to the phosphate group (PO_4_^3−^) in the HAp. The dentin powder treated with the S-PRG eluate for 3 h and the untreated dentin (control) showed three major vibrational band peaks centered at 1032, 603, and 564 cm^−1^, again corresponding to the phosphate group (PO_4_^3−^) in the hydroxyapatite. Other two vibrational band peaks centered at 1400 and 1450 related to carbonate groups (CO_3_^2^) within the HAp^28^ and the Amide I band displayed at 1647 cm^−1^ for dentin samples. The FTIR spectra for the S-PRG fillers demonstrated two major vibrational band peaks centered at 1067 and 1375, corresponding to BO_4_ and BO_3,_ respectively^29^. In the FTIR spectra for the treated enamel and dentin specimens, it was difficult to identify the B(OH)_4_^−^ ions because their vibrational bands overlapped with those of phosphate ion (PO_4_) located in the HAp crystals.

### pH measurement

**Figure 3:**
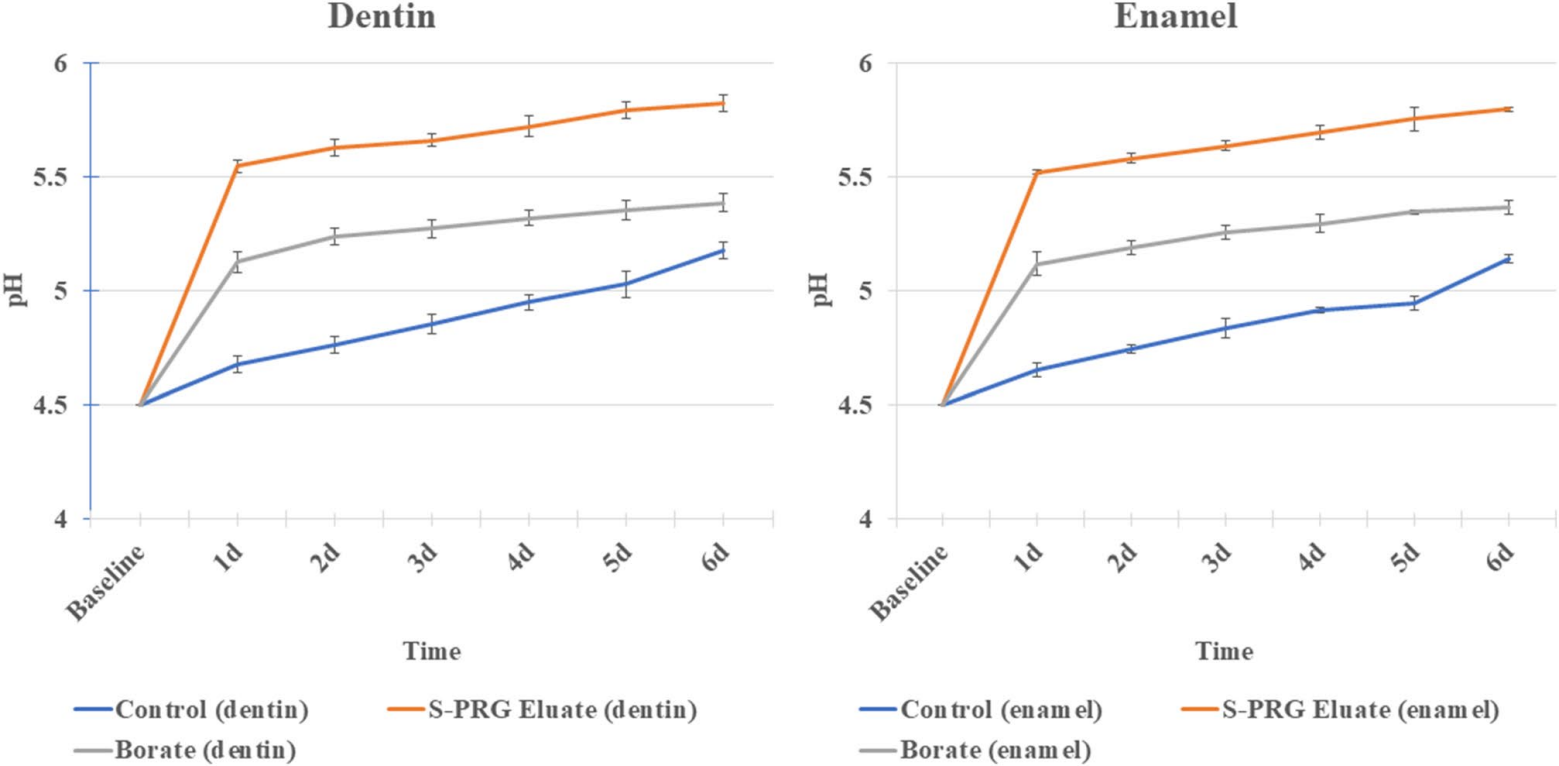
The pH plotted against time showing the changes in the pH values for the different tested groups. Statistical analysis was performed using two-way repeated measures ANOVA.

The pH value of the solution (Table 2), containing untreated dentin (control), gradually and slightly increased from 4.68 (± 0.03) after 1 day to reach 5.18 (± 0.03) after 6 days. In the case of the solution containing dentin treated with S-PRG eluate for 3 h, the pH value showed a rapid increase to 5.54 (± 0.02) after 1 day and a further increase to reach 5.82 (± 0.01) after 6 days. For the solution containing dentin samples treated with borate solution for 3 h, the pH value showed a slight rapid increase, reaching 5.13 (± 0.04) after a day, followed by an increase reaching 5.38 (± 0.02) after 6 days. The pH values of the S-PRG and borate groups were significantly higher than those of the control group at all time intervals with p < 0.05 (Fig. 3). The pH values of the solutions containing enamel samples showed the same pattern as the dentin samples, but with slight lower degree of increase; the solutions of the control group showed a gradual increase in the pH value, starting from 4.66 (± 0.03) after a day and reaching 5.14 (± 0.02) after 6 days. The S-PRG eluate group showed a rapid increase in pH value reaching 5.52 (± 0.01) after a day, followed by a slight gradual increase in pH value until it reached 5.80 (± 0.01) after 6 days. The borate group solution showed a rapid increase in pH to 5.12 (± 0.05) after a day, followed by a stable slight increase to 5.37 (± 0.03) after 6 days. pH values of S-PRG eluate and borate group were significantly higher than those of the control group at all time intervals at p < 0.05 (Fig. 3).

**Table 2:** Means and standard deviation for the pH values of the solutions containing the different tested groups (A) Dentin, (B) Enamel.

	Control	Eluate	Borate
**(A)**			
Day 1	4.68 ± 0.03^aA^	5.55 ± 0.02^aB^	5.13 ± 0.04^aC^
Day 2	4.76 ± 0.01^bA^	5.63 ± 0.01^bB^	5.24 ± 0.02^bC^
Day 3	4.85 ± 0.01^cA^	5.66 ± 0.02^cB^	5.27 ± 0.02^cC^
Day 4	4.95 ± 0.01^dA^	5.72 ± 0.01^ dB^	5.32 ± 0.01^dC^
Day 5	5.03 ± 0.06^eA^	5.79 ± 0.01^eB^	5.35 ± 0.01^eC^
Day 6	5.18 ± 0.03^fA^	5.82 ± 0.01^eB^	5.38 ± 0.02^eC^
**(B)**			
Day 1	4.66 ± 0.03^aA^	5.52 ± 0.01^aB^	5.12 ± 0.05^aC^
Day 2	4.75 ± 0.01^bA^	5.58 ± 0.02^bB^	5.19 ± 0.02^bC^
Day 3	4.84 ± 0.04^cA^	5.64 ± 0.01^cB^	5.26 ± 0.01^cC^
Day 4	4.92 ± 0.01^dA^	5.7 ± 0.03^ dB^	5.3 ± 0.04^cC^
Day 5	4.95 ± 0.01^dA^	5.75 ± 0.05^eB^	5.35 ± 0.01^dC^
Day 6	5.14 ± 0.02^eA^	5.8 ± 0.01^fB^	5.37 ± 0.03^dC^

Different superscript uppercase letters indicate significant difference within each row, and lowercase letters indicate significant difference within each column (p < 0.05).

### FE-SEM observation

**Figure 4:**
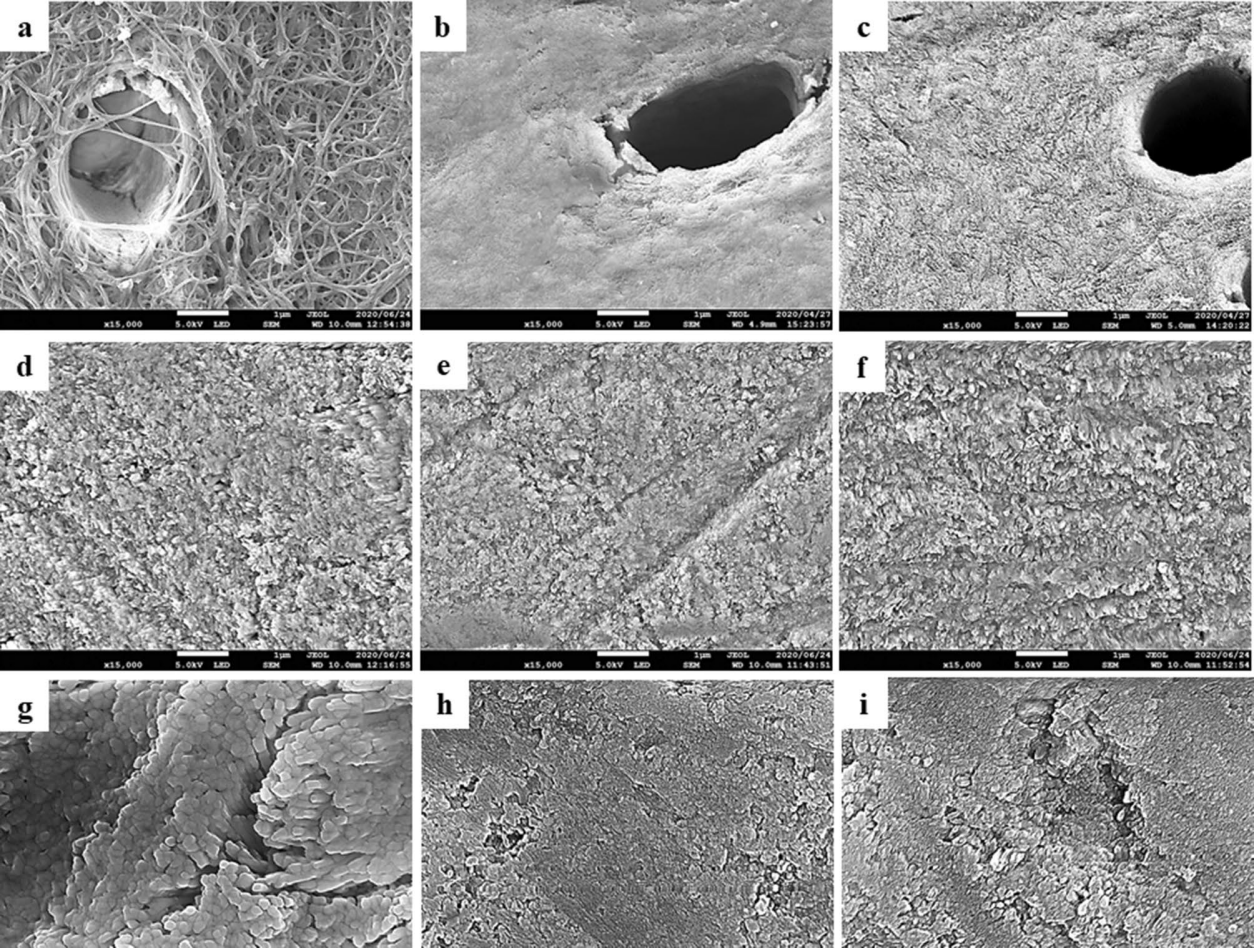
SEM images for the dentin and enamel surfaces at ×15,000 magnification (scale bar 1 µm), (**a**) control “dentin”, (**b**) S-PRG eluate “dentin”, (**c**) Borate “dentin”, (**d**) control “enamel”, (**e**) S-PRG eluate “enamel”, (**f**) Borate “enamel”, (**g**) control “enamel” at ×30,000 magnification while arrows refer to the interprismatic area (scale bar 0.1 µm), (**h**) S-PRG eluate “enamel” at ×30,000 magnification (scale bar 0.1 µm), (**i**) Borate “enamel” at ×30,000 magnification (scale bar 0.1 µm).

The FE-SEM images in the untreated dentin specimens (control) (Fig. 4a) revealed a demineralized dentin surface associated with exposure of dentin collagen. The images in the dentin specimens treated with the S-PRG eluate for 3 h (Fig. 4b) showed a slight demineralization of the peritubular dentin, but apparently an intact dentin surface. The dentin sample treated with the borate solution for 3 h (Fig. 4c) showed the similar morphology as the eluate of S-PRG, but there was a trace of erosion in the intertubular dentin in this sample.

All enamel specimens did not show any prismatic pattern (Fig. 4d–f), indicating that the surfaces were not demineralized (Supplemental figure S1). However, in the control enamel, an array of enamel crystals was observed in the area between the prisms in the high magnification (30,000×) image (Fig. 4g). On the other hand, the S-PRG eluate group (Fig. 4e,i) and the borate group (Fig. 4f,h) showed an intact enamel surface and less exposure of enamel crystals.

## Discussion

Solid-state (SS) MAS-NMR spectroscopy revealed the interaction of borate ions released from S-PRG fillers with the biological apatites of enamel and dentin. The results showed that when the S-PRG eluate interacted with enamel and dentin, tetra-coordinated borate ions B(OH)_4_^–^ were adsorbed on HAp, whearas the tri-coordinated borate B(OH)_3_ was not located at any site in the enamel or dentin substrate.

The behavior of borate ions released from S-PRG was not completely understood. A previous ICP-AES analysis revealed the effective incorporation of borate ions into the human dental enamel through the simultaneous integration of anions and cations under balanced-charge circumstances^19^. However, this study did not determine the specific borate species incorporated.

We performed the ^11^B NMR analysis on enamel and dentin, providing evidence of the coordination mode of borate ion. The difference in NMR spectra between the BO_3_ unit and BO_4_ unit is due to the structural differences between both units. The BO_3_ unit consists of a planer structure, in which the boron lies at the center of an oxygen triangle (trigonal). Therefore, the nuclear quadrupole moment of ^11^B in BO_3_ unit becomes large. Alternatively, in the BO_4_ unit, the boron is located at the center of an oxygen tetrahedron (tetrahedral). The quadrupole moment becomes small due to the spherical symmetry around the ^11^B, hence the a sharper resonance line is observed for the transition. These two specific ^11^B adsorptions from BO_3_ and BO_4_ can easily be separated, enabling one to obtain the fraction of each species^30^.

Boric acid is a very weak acid with a pKa value of 9.24. This acid accepts a pair of electrons from a hydroxyl ion to form a borate ion, acting as a Lewis acid. The dominant forms of boron atoms in aqueous systems exist as either non-ionized tri-coordinated B(OH)_3_ or ionized tetra-coordinated B(OH)_4_^−^^29, 31^, exchanging with each other in the state of equilibrium within the aqueous media^32, 33^ as below:

1 $${\text{B}}\left( {{\text{OH}}} \right)_{{3}} + {\text{H}}_{{2}} {\text{O}} \rightleftarrows {\text{B}}\left( {{\text{OH}}} \right)_{{4}}^{ - } + {\text{H}}^{ + } {\text{pKa}} = {9}.{24}$$

Thus, the status of borate ions is affected by the pH value of the solution^31^. Since the eluate of S-PRG was at pH 7, which was lower than the pKa value, B(OH)_3_ should have been predominant and the eluate should have contained only trace amounts of ionized B(OH)_4_. This was confirmed by the NMR results, which showed that non-ionized B(OH)_3_ was predominant in the eluate (Fig. 1b), and the signal of ionized B(OH)_4_ was rarely detected.

However, the B(OH)_4_^−^ was detected in the enamel and dentin samples treated with the eluate and the nano-HAp “pure inorganic apatite” specimens. This finding might be related to the electrostatic adsorption of the negatively charged B(OH)_4_^−^ ions occurring on the sites of positively charged calcium ions in the enamel, dentin, and nano-HAp substrates^34^. The continuous adsorption of the B(OH)_4_^−^ ions to the positively charged sites would shift the equilibrium between B(OH)_3_ and B(OH)_4_^−^ ions to the right side of the Eq. (1), favoring the formation of more B(OH)_4_^−^ ions. Thus, the consumption of B(OH)_4_^−^ ions due to the electrostatic adsorption on enamel and dentin surfaces produced additional B(OH)_4_^−^ ions. This electrostatic adsorption of B(OH)_4_^−^ ions might continue until the surface of enamel, dentin and nano-HAp substrates were electrostatically neutralized.

The electrostatic adsorption site of the B(OH)_4_^−^ ions with HAp can be possible because of the charge distribution of the HAp hexagonal structure. This structure of the HAp crystal possesses two planes. a(b)-plane mainly presents positively-charged calcium ions, while c-plane mainly presents negatively-charged phosphate ions and hydroxyl groups^35^. In the case of enamel, the presence of amelogenin “enamel matrix protein” adjacent to the a- and b-surfaces of the HAp crystallites favors the crystallite growth in the c-axis direction resulting in thin and long crystals^36^. Whereas in the dentin HAp case scenario, the arrangement of nano-crystalline clusters of amorphous calcium phosphate support the crystallite growth and elongation in the c-axis direction, leading to an increase in the a(b)-plane^37^. Consequently, this may render the HAp to become more positively charged. Moreover, the incorporation of the CO_3_^2−^ ions in the hexagonal apatite structure in dentin induces the increase of the positively-charged calcium-rich a(b)-planes of the HAp crystal leading to more adsorption of the B(OH)_4_^−^ ions^35^. Therefore, the difference in crystallinity and composition between dentin and enamel may be related to the reactivity of borate ions to dentin and enamel. In fact, the spectral peak of dentin was more intense than that of enamel, indicating a higher concentration of borate ions in dentin. This suggests that dentin is more reactive than enamel to borate ions.

Regarding the FTIR spectra (Fig. 2), the relationship between the infrared spectra and the molecular configuration for the boric acid (BO_3_) and borate ion (B(OH)_4_^−^) was clarified by Nakamoto et al. using symmetry rules, defining boric acid as YX3 and borate ion as YX4^38^. Considering this relation, boric acid (YX3), with its asymmetric B–O stretching (v3), should have one IR active peak (1500–1300 cm^−1^), while borate ion (YX4) has one broad v3 asymmetric stretching band with an active IR peak around 950 cm^−1^^38^. The difficulty in identifying B(OH)_4_^−^ ions in the enamel and dentin specimens might be attributed to the overlap between the PO_4_ and BO_4_ peaks. It is, however, suggested that trigonal boron might not present in the enamel and dentin specimens. Meanwhile, BO_3_ peak was only found in the S-PRG filler, confirming the NMR results.

In the pH measurements (Fig. 3), the rapid increase in the pH measurement values within the first day in the S-PRG and borate groups might be related to the release of ions in large quantities during this short period compared with the other periods^25^. The control groups (enamel and dentin), buffered the demineralizing solution as a result of ionic dissolution during enamel and dentin demineralization and subsequent release of Ca_2_^+^, OH^−^ and PO_4_^3+^, resulting in neutralization of the pH^25^. Besides this original buffering effect of HAp, the S-PRG treated groups possessed additional buffer capacity as the adsorbed tetrahedral borate ions on the surface of the treated HAp are more susceptible to be converted to trihedral in acidic (protonated) media rather than in neutral or basic media^39^. When the conversion of tetrahedral boron to trigonal boron occurred, acid (hydrogen ions) was consumed, which subsequently increased the pH^39^. This ability to consume hydrogen ions (H^+^) gives the borate solution its buffer capacity^40^, as shown in Eq. (1).

Besides the borate ion, it was suggested that the buffering capacity of S-PRG also resulted from the release of ions into a solution such as Sr^2+^ and Na^+^ by an ion exchange process for H^+^ ions and the formation of alkaline compounds, such as NaOH and Sr(OH)_2_^4^. In addition, the release of fluoride ions can exchange with the H^+^ ions in the solution, rendering it more alkaline. Also, fluoride ions can induce the precipitation of apatites from acidic precursors, such as amorphous calcium phosphate and octacalcium phosphate^41^. Thus, the multiple ions are involved in the buffer capacity of S-PRG. For these reasons, the eluate of S-PRG showed a greater buffering effect than the borate solution.

The buffer capacity of the S-PRG and borate solutions were confirmed by FE-SEM images (Fig. 4) which revealed a more intact dentin surface than the demineralized dentin surface in the control group that showed an exposed collagen matrix. The slight morphological difference between the S-PRG and the borate samples might be related to the higher pH value for the S-PRG sample than the borate sample.

Prisms typically appear on the demineralized enamel surface as observed by SEM, while prisms do not appear on intact ground enamel^42^. However, the in current study, no remarkable prismatic pattern was observed for all enamel samples. This might be related to the fact that the surface was moderately demineralized due to the use of pH 4.5 solution for 6 days without replacement. When observed at high magnification (30,000×), the untreated control group showed signs of demineralization with an array of enamel crystals (Fig. 4g), confirming the morphological differences from the treated groups. The difference in the SEM images was consistent with that in the increase in pH, showing a slower pH increase for the control than the treated groups.

In the present investigation, it was found that the S-PRG eluate mainly contained aqueous B(OH)_3_, but negatively charged B(OH)_4_^−^ ions were adsorbed on the surface of enamel and dentin. The adsorbed B(OH)_4_^−^ ions might be used to neutralize acidic conditions when B(OH)_3_ was converted to B(OH)_4_^−^. Further studies are needed to focus on the quantitative information on the degree of dentin demineralization.

## Conclusion

Borate ions adsorbe to the surface of enamel and dentin powder in the tetra-coordinated form. The adsorbed B(OH) _4_^−^ ions possess a buffer capacity when B(OH)_3_ converted to B(OH)_4_^−^, which helps to protect the tooth structure against acid attacks.

## Supplementary Information

Supplementary Information 1.
