# Supplementary material for: Interactions of boron released from surface pre-reacted glass ionomer with enamel/dentin and its effect on pH
Source: Sci Rep. 2021 Aug 3;11:15734. doi: 10.1038/s41598-021-95279-x (PMC8333262; doi:10.1038/s41598-021-95279-x)

## Slide 1
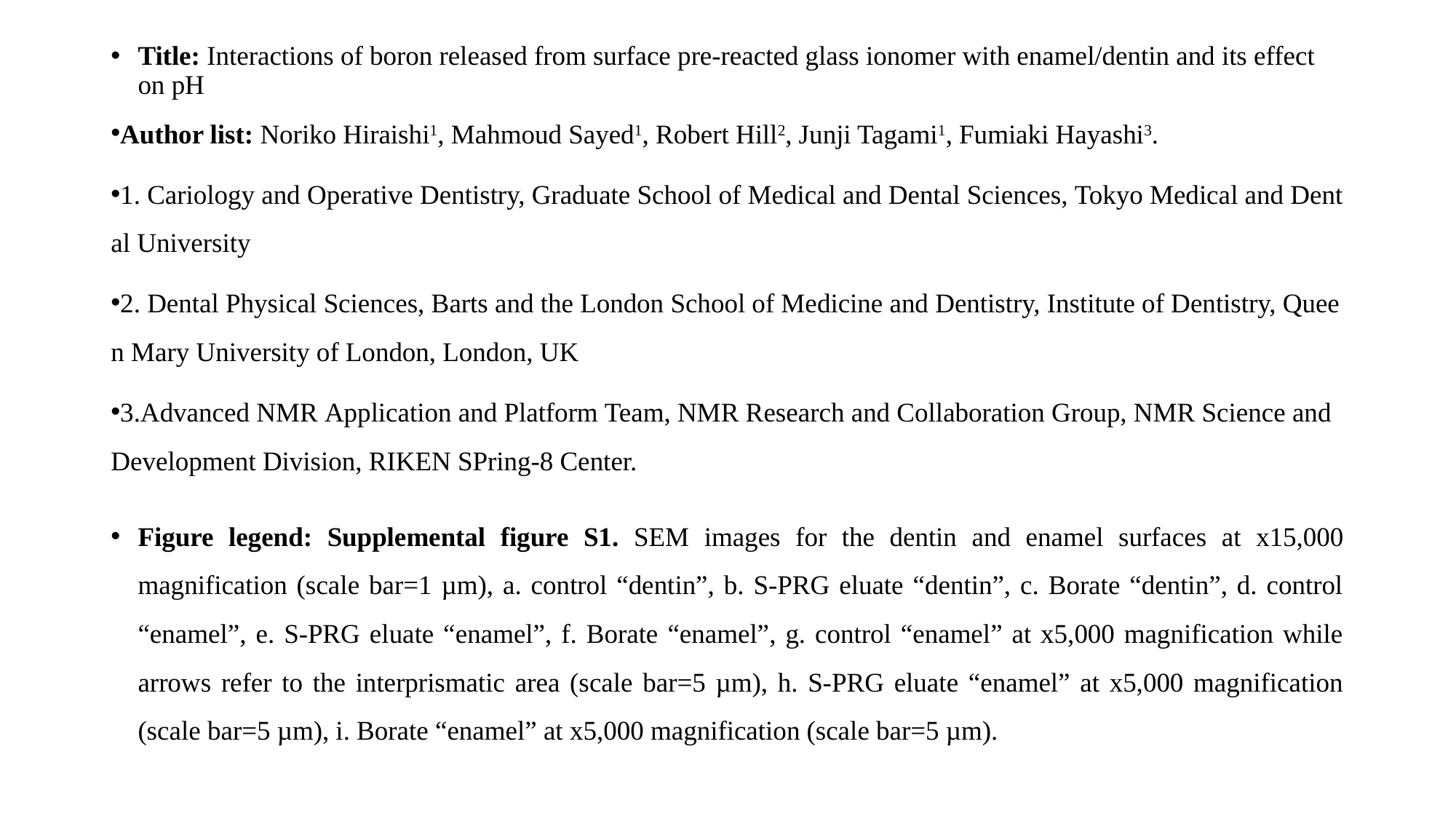

Title: Interactions of boron released from surface pre-reacted glass ionomer with enamel/dentin and its effect on pH
Author list: Noriko Hiraishi1, Mahmoud Sayed1, Robert Hill2, Junji Tagami1, Fumiaki Hayashi3.
1. Cariology and Operative Dentistry, Graduate School of Medical and Dental Sciences, Tokyo Medical and Dental University
2. Dental Physical Sciences, Barts and the London School of Medicine and Dentistry, Institute of Dentistry, Queen Mary University of London, London, UK
3.Advanced NMR Application and Platform Team, NMR Research and Collaboration Group, NMR Science and Development Division, RIKEN SPring-8 Center.
Figure legend: Supplemental figure S1. SEM images for the dentin and enamel surfaces at x15,000 magnification (scale bar=1 µm), a. control “dentin”, b. S-PRG eluate “dentin”, c. Borate “dentin”, d. control “enamel”, e. S-PRG eluate “enamel”, f. Borate “enamel”, g. control “enamel” at x5,000 magnification while arrows refer to the interprismatic area (scale bar=5 µm), h. S-PRG eluate “enamel” at x5,000 magnification (scale bar=5 µm), i. Borate “enamel” at x5,000 magnification (scale bar=5 µm).

## Slide 2
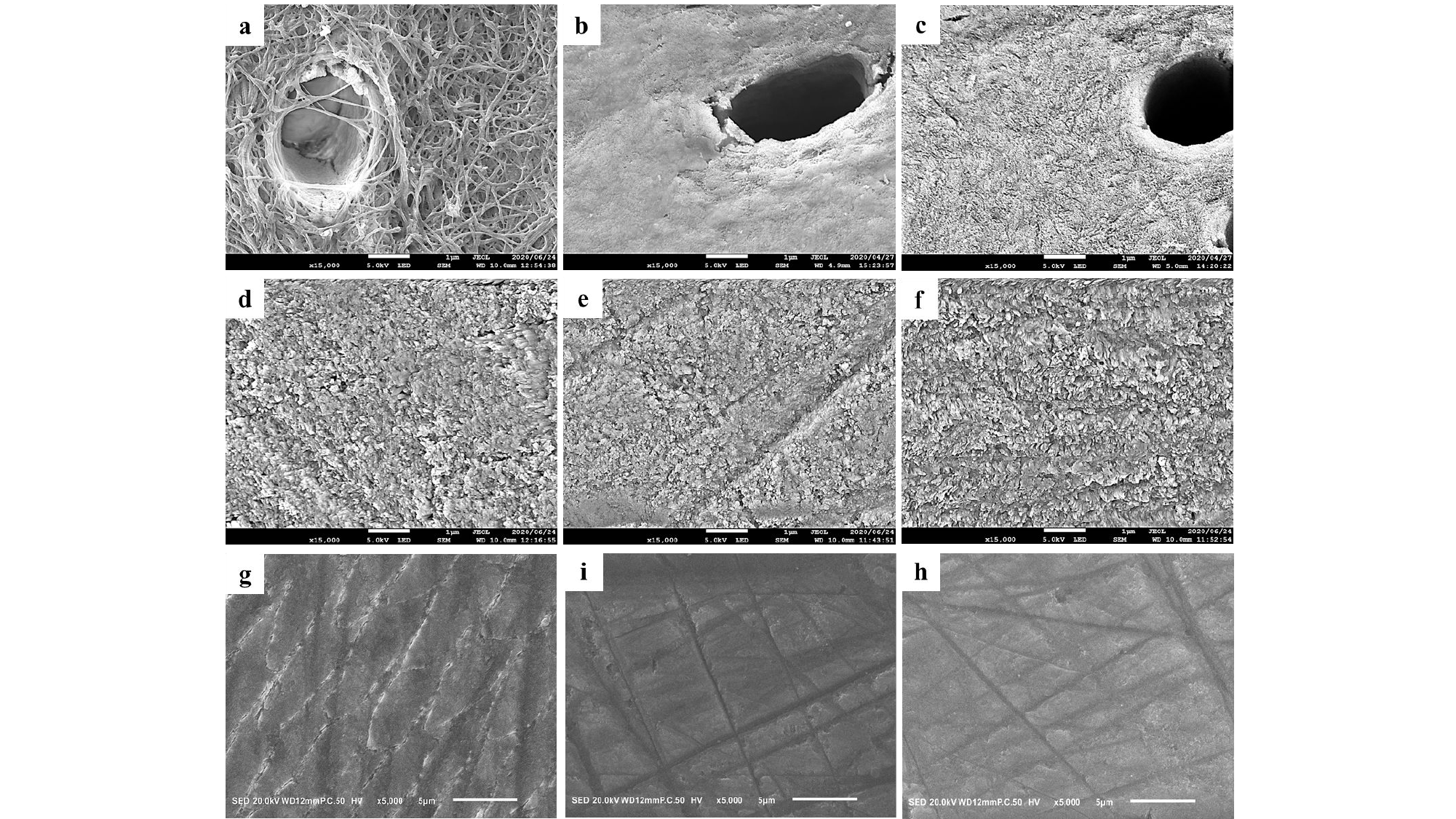

Supplement: Supplementary file 1 — Supplementary Information 1. [file 41598_2021_95279_MOESM1_ESM.pptx]
